# A parallel convolution attention network for crop genomic prediction

**DOI:** 10.1371/journal.pone.0358451

**Published:** 2026-09-21

**Authors:** Wenyu Peng, Yuxia Sheng, Ying Zhou

**Affiliations:** 1 School of Electronic Information, Wuhan University of Science and Technology, Wuhan, China; 2 College of Life Science and Health, Wuhan University of Science and Technology, Wuhan, China; Shandong Agricultural University, CHINA

## Abstract

Genome prediction is a breeding strategy that predicts crop phenotype through genomic markers. Traditional statistical models cannot accurately capture the complex relationships between genotypes and phenotypes, and deep neural networks have shown significant advantages in capturing nonlinear relationships. Deep learning methods in genome selection primarily utilize sequential convolutional kernels, which can lead to challenges such as gradient disappearance and computational inefficiency. In this study a novel crop genomic prediction method based on a parallel convolutional attention network (PCAGP) is proposed. The constructed deep learning framework uses the convolution kernel to process information through a parallel structure, combined with a coordinate attention mechanism that effectively integrates inter-channel relationships and spatial genomic information. PCAGP was compared with eight widely used genomic prediction methods on four benchmark datasets, including a traditional statistical method, three machine learning methods, and four popular deep learning methods. The experimental results demonstrate that PCAGP outperforms all baseline methods and significantly improves genomic prediction accuracy.

## Introduction

As uncertainty about climate change intensifies, food security challenges are becoming increasingly severe. Threats to crop yield and stability, such as extreme weather events, rising pest and disease pressures, and the degradation of arable land, continue to escalate [1]. The development of high-yield, disease-resistant crop varieties is therefore essential, both to mitigate land overuse and address the projected sharp rise in food demand [2]. By enabling more effective selection of superior agronomic traits, accurate trait prediction accelerates the deployment of new varieties to combat food scarcity [3]. Genomic prediction enables the estimation of breeding values based on genome-wide molecular markers, thereby assisting breeders in making informed selection decisions [4].

Genomic prediction is the first step in genomic selection (GS) for plant breeding programs. GS is an advanced breeding technology widely used in animal and plant breeding with significant potential [5]. It utilizes genome-wide marker information to predict the genetic potential of individuals. Compared to traditional breeding methods, GS does not require the identification of specific loci significantly associated with target traits. Instead, it captures the subtle effects of each locus in the genome through high-density genetic markers [6]. This ability gives GS obvious advantages in capturing the genetic effects of complex traits, effectively improving crop yield and disease resistance. However, the effectiveness of GS is influenced by various factors, including the size of the training population, the heritability of traits, marker density, and the predictive methods employed [7,8].

Traditional statistical methods, such as the genomic best linear unbiased prediction (GBLUP) model [9] and bayesian approaches [10], have been widely used to model genotype effects and predict phenotypes. However, these models typically assume that the random effects of genotypes follow a certain prior distribution. In practical applications, the effects of genotypes are often unknown, and it is challenging to capture complex non-additive effects [11]. For example, the GBLUP model assumes that all markers contribute equally to the relationship matrix, which may not be the case in reality. To address these limitations, several improved GBLUP-based methods have been developed. The GA-GBLUP method introduces a genetic algorithm (GA) to optimize marker weights, thereby selecting markers associated with the target trait [12]. The multi-trait GBLUP model, which integrates global or local genetically relevant information, has also been used to improve the accuracy of multi-population genomic prediction [13]. Furthermore, the application of the L2,1 norm-regularized multiple regression model in genomic selection provides new ideas for more efficient multi-trait prediction [14]. Beyond classical statistical approaches, machine learning methods such as random forest (RF), support vector regression (SVR), and extreme gradient boosting (XGBoost) have been applied to the prediction of crop agronomic traits [15,16]. The accuracy and variability of machine learning methods, including neural networks, were evaluated in genomic prediction analyses using simulated traits. Although these methods excel at capturing non-linear relationships, their performance can be constrained when handling high-dimensional and complex genomic datasets, highlighting the need for more advanced computational frameworks.

In recent years, deep learning (DL) methods have gained popularity in GS, facilitating the application of large-scale genomic data in phenotypic prediction [17]. Researchers are able to more precisely identify genetic markers associated with traits, thereby accelerating traditional breeding processes and reducing costs [18]. Among these, convolutional neural networks (CNNs) have shown significant advantages in the field of genomic selection, particularly in capturing complex nonlinear relationships and gene interactions. Genomic prediction model based on deep learning framework, such as, DNNGP [19], SoyDNGP [20],Cropformer [21], MeNet [22], CLCNet [23], and WheatGP [24], have made significant progress in crop breeding. In addition, genome prediction methods based on genotype–environment interactions have also achieved substantial improvements, including the gated multilayer perceptron and linear attention–integrated model (GEFormer) [25]. However, most deep learning models all rely on serial-structured convolution kernels to extract features, which can lead to information loss when analyzing complex relationships within genomic data. Additionally, determining the optimal size of the convolution kernel remains a time-consuming and computationally intensive task. In contrast, a parallel convolutional structure extracts features simultaneously using multiple convolution kernels of different sizes, reducing the need for hyperparameter tuning. This improves the expressiveness and diversity of the extracted features [26]. Moreover, this structure has been shown to enhance computational efficiency while preserving fine-grained details.

In this study, we construct a novel crop genomic prediction method based on a parallel convolutional attention network (PCAGP). The PCAGP model consists of a parallel convolutional layer, a coordinate attention (CA) layer, and two fully connected (FC) layers. The parallel convolutional layer simultaneously extracts genotype features using convolution kernels of different sizes. The CA layer effectively captures long-range dependencies while preserving precise positional information. The FC layers perform global feature fusion and extraction through linear transformations and nonlinear activation functions. To evaluate the performance of the PCAGP method, we conducted comprehensive benchmark experiments on datasets of soybean, wheat, and rice. The results demonstrate that, compared to six widely used prediction models, PCAGP achieves superior prediction accuracy for agronomic traits across different crop species.

## Materials and methods

### PCAGP model structure

**Fig 1:**
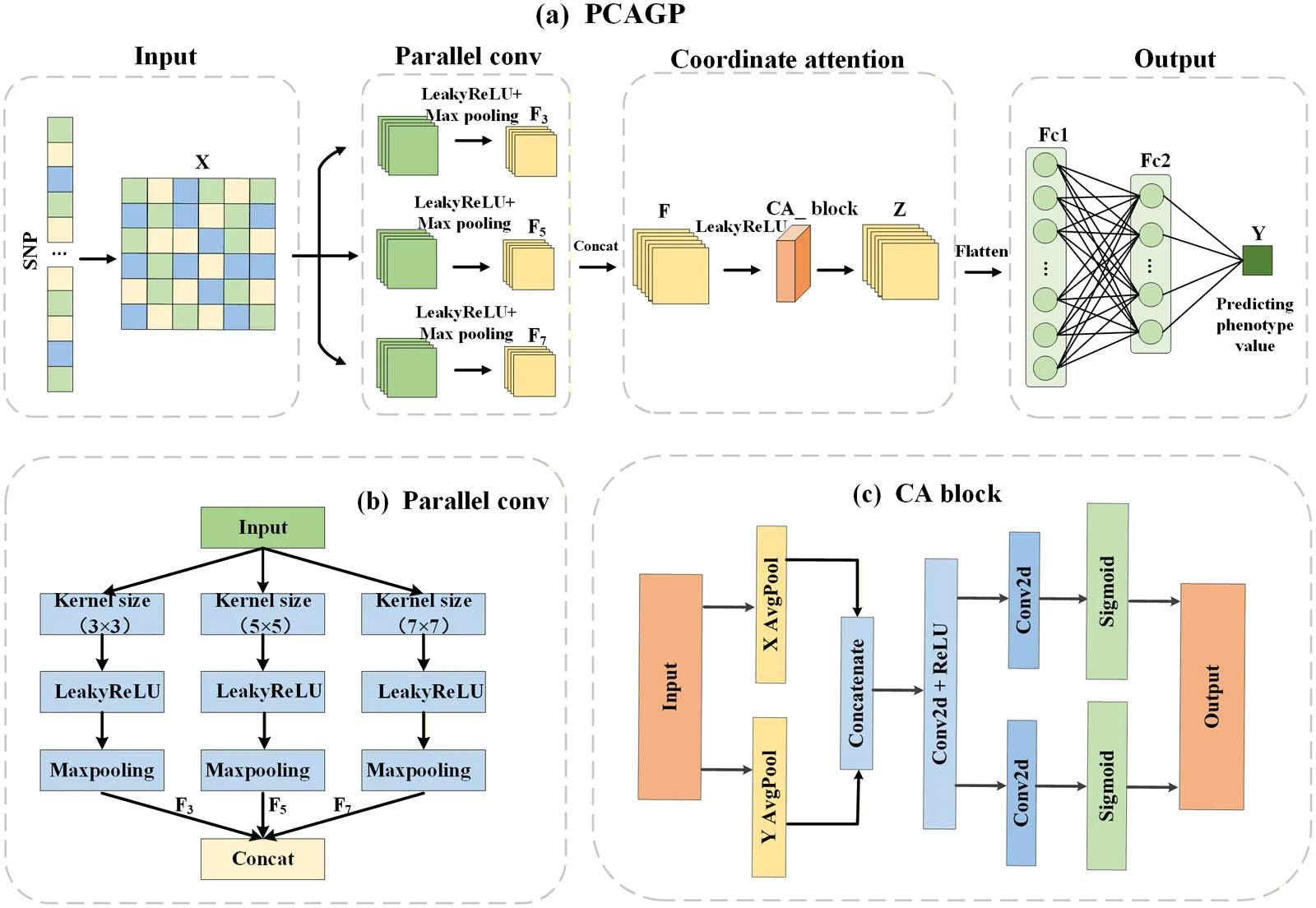
The architecture of PCAGP. (**a**) The flow chart of PCAGP. This model converts the SNP loci corresponding to each sample into an $\left\lceil \sqrt{N}\right\rceil \times \left\lceil \sqrt{N}\right\rceil$ genotype matrix in the input layer. Then, through the parallel convolutional layer, three convolution kernels (3 × 3, 5 × 5, 7 × 7) are used for feature extraction. Next, through the coordinate attention layer, the inter-channel and spatial location information are effectively utilized. Finally, through the fully connected layer, the predicted value of the phenotype is output. (**b**) Parallel convolution module. (**c**) Block diagram of the CA attention module. Firstly, the input data is average pooled in horizontal and vertical directions. Then, the pooled data is concatenated in the channel dimension. Next, 1 × 1 convolution is used to compress the number of channels, and the complete feature vector is re-divided into horizontal and vertical direction vectors, and then the number of channels of the feature vectors in the two directions is readjusted by 1 × 1 convolution. Finally, the Sigmoid function is used to weight the original input data in two directions.

The proposed PCAGP model consists of a parallel convolutional layer, a coordinate attention mechanism, and two fully connected layers. The parallel structure convolutional layer is used to increase the width of the model and minimize the loss of information. The coordinate attention mechanism is a fusion of location and channel information. The architecture of PCAGP is shown in Fig 1. This model performs feature extraction through a parallel convolution module, which can extract information from original data more effectively, as shown in Fig 1(b). Moreover, a coordinate attention mechanism is added after convolution to assign different weights to the features, as shown in Fig 1(c).

The input of the PCAGP model is the label encoding of the genotype data after screening. The SNP loci are converted into a matrix of size $\left\lceil \sqrt{N}\right\rceil \times \left\lceil \sqrt{N}\right\rceil$ as the network input X, where N denotes the number of SNP loci corresponding to each sample and the symbol ⌈ ⌉ represents the ceiling function. Specifically, we choose the square number ${\left\lceil \sqrt{N}\right\rceil }^{2}$ as the target dimension, pad each feature vector to length ${\left\lceil \sqrt{N}\right\rceil }^{2}$, and then reshape it into a $\left\lceil \sqrt{N}\right\rceil \times \left\lceil \sqrt{N}\right\rceil$ matrix.

The input firstly passes through three parallel convolutional layers, each of which uses convolution kernels of different sizes, namely 3 × 3, 5 × 5, and 7 × 7, to capture the different-scale features of the input data. Specifically, the first convolutional layer uses 3 × 3 kernels with a stride of 1 and padding of 1. The second convolutional layer uses 5 × 5 convolution kernels with a stride size of 1 and padding of 2. The third convolutional layer uses 7 × 7 convolution kernels with a stride size of 1 and padding of 3. Each convolution module expands the feature maps from 1 input channel to 16 output channels. The different padding sizes ensure consistent output dimensions. A 4 × 4 maxpooling layer is then applied to downsample the feature maps. The outputs of the three convolutional paths are concatenated along the channel dimension to form a unified feature matrix 𝐅. A dropout layer (dropout rate = 0.2) and the Leaky ReLU activation function are applied to enhance non-linear representation and mitigate overfitting.


$$\begin{array}{c} 𝐅=\text{Concat}({𝐅}_{3},{𝐅}_{5},{𝐅}_{7}) \end{array}$$ (1)

where ${𝐅}_{3}$, ${𝐅}_{5}$, and ${𝐅}_{7}$ represent the outputs of parallel convolution operations. The operator Concat(·) denotes channel-wise concatenation.

The concatenated feature map is then fed into the coordinate attention (CA) module to generate directional attention weights. The feature map is reweighted to enhance the representation of informative features. The output is expressed as Z.


$$\begin{array}{c} 𝐙=\text{CA}(𝐅) \end{array}$$ (2)

where CA(·) represents the textcoordinate attention mechanism.

Finally, the attention-enhanced feature map is flattened into a 1D vector, which is passed through two fully connected layers to output the predicted phenotype value Y.


$$\begin{array}{c} 𝐘=\sigma \left({𝐖}_{2}\cdot \sigma \left({𝐖}_{1}\cdot \text{Flatten}(𝐙)+{𝐛}_{1}\right)+{𝐛}_{2}\right) \end{array}$$ (3)

where σ(·) represents activation function, ${𝐖}_{1}$, ${𝐖}_{2}$ represent learnable weight matrices, and ${𝐛}_{1}$, ${𝐛}_{2}$ represent bias vectors. Flatten(·) represents the flattening operation.

The CA block is a module based on coordinate attention, designed to enhance the model’s spatial feature representation capability [27]. Its design integrates attention mechanisms in both the horizontal and vertical directions, while also considering the information between feature channels. The module first extracts global information in the horizontal and vertical directions through two adaptive average pooling operations. The two feature maps are then concatenated along the channel dimension. An 1 × 1 convolutional layer, followed by batch normalization (BN) and a ReLU activation function, is applied to reduce the dimensionality. Afterwards, the dimension-reduced features are fed into two separate 1 × 1 convolutional layers, which generate horizontal and vertical attention weights through the Sigmoid function. Finally, the horizontal and vertical attention weights are multiplied by the original feature maps to obtain the weighted output features.

The PCAGP model incorporates a batch normalization (BN) layer [28]. By normalizing inputs to a zero-mean and unit-variance distribution, BN helps prevent overfitting and gradient instability, while also accelerating training convergence. For a given feature *x*, the batch normalization formula is as follows:


$$\begin{array}{c} \hat{x}=\frac{x-\mu }{\sqrt{\delta^{2}+\beta }} \end{array}$$ (4)

where μ represents the mean of the features, $\delta^{2}$ denotes variance of the features, and β represents a small constant added to prevent division by zero.

We use the LeakyReLU activation function [29], which is an improved version of the ReLU activation function. By allowing a small, non-zero gradient when the input is negative, LeakyReLU preserves information flow in the negative domain and alleviates the vanishing gradient problem. The expression for the LeakyReLU activation function is as follows:


$$\begin{array}{c} \text{LeakyReLU}(x)=\left\{ \begin{array}{cc} x, & \text{if }x>0,\\ \alpha x, & \text{if }x\leq 0. \end{array}\right. \end{array}$$ (5)

where α is a small constant, typically set to 0.01.

Due to the challenge of the problem of a large number of SNPs and a small sample size in GS. This can lead to an overfitting issue that affects the stability and predictive performance of the model. Regularization is a common solution in machine learning to prevent overfitting. Its core function is to limit the degree of freedom of the model parameters by adding additional penalty terms. It improves the generalization ability, enhances the stability of the model, and realizes feature selection to some extent. In the PCAGP model, to mitigate the impact of outliers on training results, we use a loss function that includes the Huber loss function [30] and an L2 regularization term. The loss function used in the PCAGP model is represented as follows:


$$\begin{array}{c} L=L_{\alpha }+\lambda {\left\|\theta \right\|}_{2}^{2} \end{array}$$ (6)

where ${\text{L}}_{\alpha }$ represents the Huber loss function, θ represents the weight vector, and λ is the regularization coefficient controlling the degree of regularization. This means that larger weights in the model are more penalized, guiding the model to prefer smaller weight values.

The Huber loss function is a combination of mean squared error (MSE) and mean absolute error (MAE), and it is more robust than MSE when there are outliers in the data. It applies MSE for small errors and MAE for large errors, providing better performance when handling outliers. The Huber loss function is expressed as follows:


$$\begin{array}{c} {\text{L}}_{\alpha }(y,\tilde{y})=\left\{ \begin{array}{cc} \frac{1}{2}(y-\tilde{y}{)}^{2}, & \text{if }|y-\tilde{y}|\leq \alpha \\ \alpha |y-\tilde{y}|-\frac{1}{2}\alpha^{2}, & \text{if }|y-\tilde{y}|>\alpha \end{array}\right. \end{array}$$ (7)

where *y* and $\tilde{y}$ represent the true value and the predicted value, respectively. α is a hyperparameter used to control the error threshold.

### Datasets

We used four real datasets to evaluate the performance of the proposed model.

The first dataset, soybean573, originates from the Yangtze-Huaihe Soybean Breeding Germplasm Population (YHSBLP) established by the National Soybean Improvement Center. It includes SNP data obtained through the simplified genome sequencing technology of RAD-seq [31]. This dataset includes genotyping information for 573 samples with a total of 61166 SNPs. In addition, it includes phenotypic data for the average weight of 100 seeds from the YHSBLP population, collected in 2013, 2014, 2017, and 2018 at the Jiangpu Experimental Station of Nanjing Agricultural University. The traits include Yield_13: average hundred-seed weight in 2013, Yield_14: average hundred-seed weight in 2014, Yield_17: average hundred-seed weight in 2017, and Yield_18: average hundred-seed weight in 2018.

The second dataset, soybean13784, originates from two integrated online databases, SoyBase and GRIN-Global [32,33]. It contains genotypic data for 20087 soybean accessions based on the SoySNP50K iSelect BeadChip platform, including 42509 high-confidence single nucleotide polymorphisms (SNPs). 13784 samples and 32032 SNPs were used for the experiments. The traits from the GRIN-Global database (https://npgsweb.arsgrin.gov/gringlobal/search) include protein content (protein), oil content (oil), hundred-seed weight (SdWgt), and yield (Yield).

The third dataset, wheat2000, consists of 2000 Iranian bread wheat landraces from the CIMMYT wheat genebank [34]. This dataset includes genotyping data for 33709 DArT markers from 2000 landraces and six agronomic traits: grain length (GL), grain width (GW), grain hardness (GH), thousand kernel weight (TKW), test weight (TW), and grain protein (GP).

The fourth dataset, rice1489, originates from the CropGS-Hub database [35]. The rice data (GSTP007) was selected. Data dimensionality was reduced using PLINK (v1.9) for linkage disequilibrium (LD) pruning (–indep-pairphase 100 10 0.2) and minor allele frequency (MAF) filtering (maf 0.05). The dataset consists of 1489 samples and 14871 SNPs. The traits include panicle number (PN), flag leaf length (FLL), flag leaf width (FLW), and panicle length (PL).

## Experiments and results

### Experimental setup and evaluation metrics

All experiments are performed on a computer equipped with an Intel i7 CPU and an NVIDIA GeForce RTX 4090 GPU. The tools used in this study are Anaconda and PyCharm. The implementation framework of PCAGP is Python 3.8.17, PyTorch 2.4.0. The learning rate is 0.00009 and the optimizer is Adam. Our training process runs for 150 epochs. We employ 5-fold cross-validation to evaluate the prediction performance of the model. For all numerical phenotypes, a linear normalization procedure was employed to constrain their values to the range [0, 1], in order to avoid the influence of different value scales on model training. The data and code are available in https://github.com/Crop-breeding/PCAGP.

In this study, we evaluated the model performance using the Pearson correlation coefficient (PCC) and mean squared error (MSE). The PCC measures the linear relationship between two variables and has a range of [−1, 1]. The prediction accuracy mentioned in this study refers specifically to PCC values.

### Comparison with other prediction methods

We compare the performance of PCAGP with eight GS methods, including one statistical method (GBLUP), three machine learning methods (RF, SVR, and XGBoost), and four deep learning methods (DNNGP [19], Cropformer [21], WheatGP [24], and MeNet [22]). We conduct experiments for different agronomic traits on four datasets. The specific implementations of these methods are as follows. GBLUP is implemented using the R packages BGLR (v1.1.3). RF is implemented in Python using scikit-learn library. SVR is implemented in Python using scikit-learn library, and rbf is adopted as the kernel function. XGBoost algorithm is implemented utilizing the xgboost library (v2.1.4) within the Python environment. For the DNNGP, Cropformer, WheatGP, and MeNet, we utilize the original public code provided by the authors, which are accessible via public links. The results are obtained using the default parameter settings provided in the original papers.

**Fig 2:**
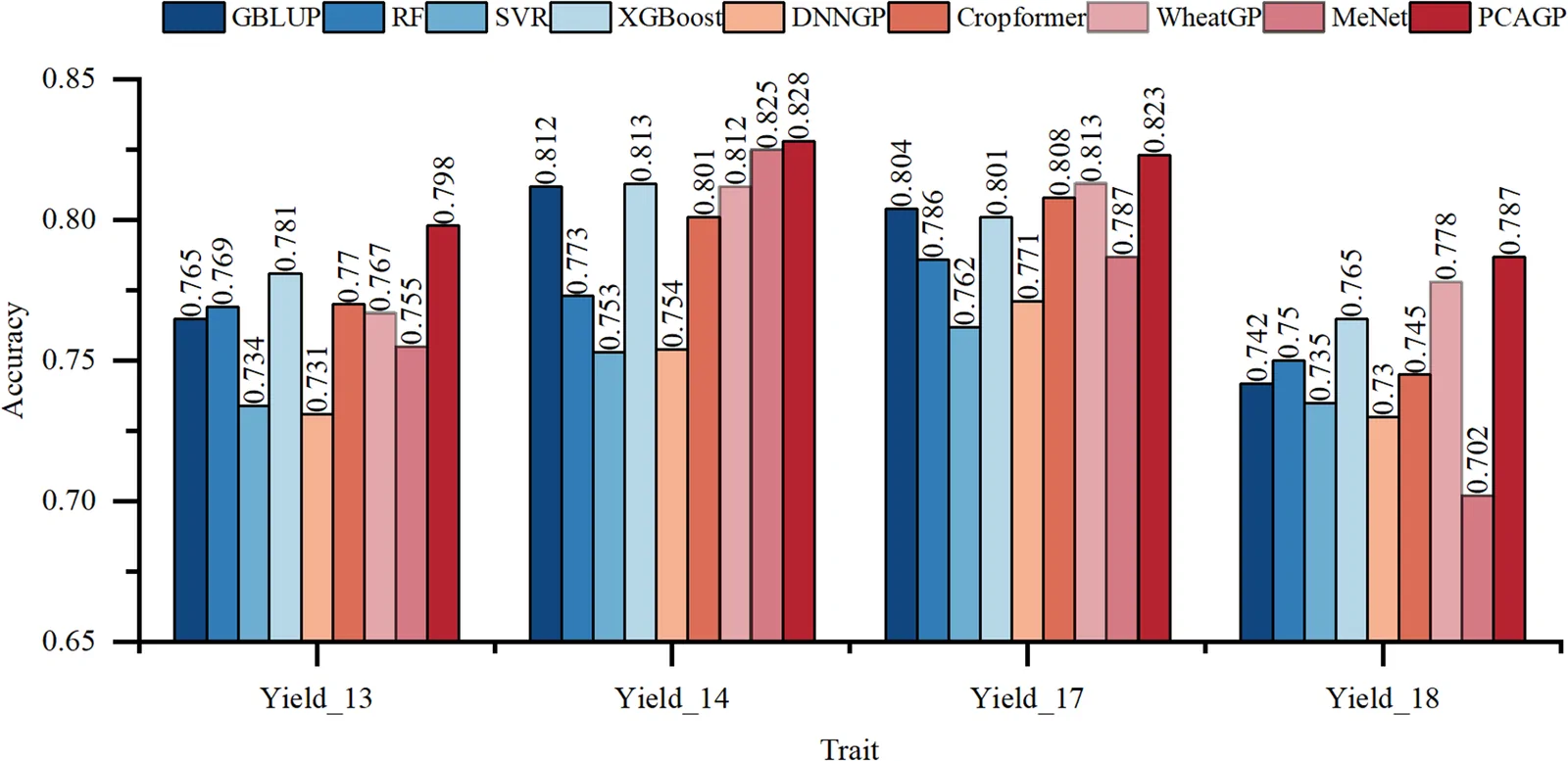
Prediction accuracy of nine methods on the soybean573 dataset. Yield_13, Yield_14, Yield_17, and Yield_18 represent average hundred-seed weight in 2013, 2014, 2017 and 2018, respectively.

As shown in Fig 2, PCAGP had the highest prediction accuracy for four traits on the soybean573 dataset.The prediction accuracy of PCAGP were improved by 4.04%, 1.97%, 1.23%, and 1.16% for four traits, respectively, with the best comparison method (WheatGP). For all traits, PCAGP had the highest average prediction accuracy (0.809), and outperformed GBLUP, RF, SVR, XGBoost, DNNGP, Cropformer, WheatGP, and MeNet by 3.6%, 5.1%, 8.5%, 2.4%, 8.4%, 3.59%, 2.08%, and 5.43%, respectively. The MSE values of different methods are shown in Table 1. PCAGP had the lowest average MSE value, 10.9%, 18.2%, 25%, 7.2%, 47.1%, 99.2%, 17.43%, and 17.43% lower than those of the comparison methods.

**Table 1:** MSE of Nine Methods on the Soybean573 Dataset.

Trait	GBLUP	RF	SVR	XGBoost	DNNGP	Cropformer	WheatGP	MeNet	PCAGP
Yield_13	0.0094	0.0100	0.0110	0.0091	0.0175	1.5356	0.0105	0.0101	**0.0082**
Yield_14	0.0074	0.0094	0.0100	0.0078	0.0150	0.6793	**0.0070**	0.0080	0.0073
Yield_17	0.0102	0.0114	0.0123	0.0101	0.0185	1.5739	0.0125	0.0116	**0.0094**
Yield_18	0.0136	0.0134	0.0137	0.0119	0.0184	0.8784	0.0136	0.0139	**0.0111**

**Fig 3:**
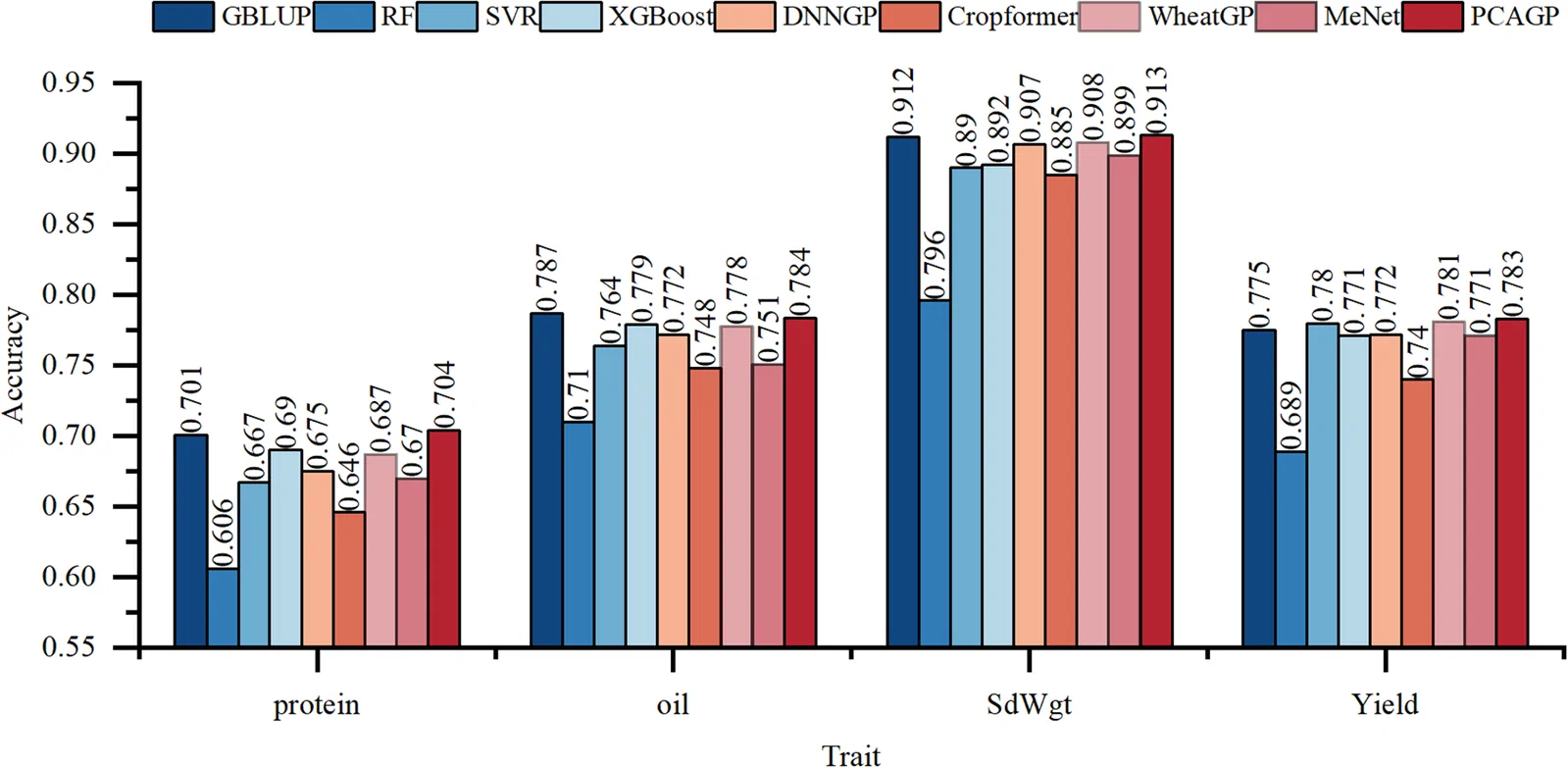
Prediction accuracy of nine methods on the soybean13784 dataset. Protein, oil, SdWgt, and Yield represent protein content, oil content, hundred-seed weight and yield, respectively.

The prediction accuracy of different methods on the soybean13784 dataset are shown in Fig 3. The SdWgt had the highest prediction accuracy for all methods. For all traits, PCAGP had the highest average prediction accuracy (0.796), and outperformed GBLUP, RF, SVR, XGBoost, DNNGP, Cropformer, WheatGP, and MeNet by 0.28%, 13.6%, 2.7%, 1.7%, 1.8%, 5.5%, 0.95%, and 3.01%, respectively. GBLUP was the second-best method (0.794). Compared with GBLUP, the prediction accuracy of PCAGP (0.784) was lower than that of (0.787) for oil, but 0.4%, 0.1%, and 1% higher for protein, SdWgt, and Yield, respectively. The MSE values of different methods are shown in Table 2. GBLUP had the lowest average MSE value. The average MSE value of PCAGP was the second lowest, being 30.9%, 8.6%, 8.6%, 92.2%, 99.1%, 3.45%, and 11.51% lower than those of RF, SVR, XGBoost, DNNGP, Cropformer, WheatGP, and MeNet, respectively.

**Table 2:** MSE of Nine Methods on the Soybean13784 Dataset.

Trait	GBLUP	RF	SVR	XGBoost	DNNGP	Cropformer	WheatGP	MeNet	PCAGP
protein	**0.0052**	0.0066	0.0057	0.0055	0.0963	0.8007	0.0055	0.0061	0.0053
oil	**0.0049**	0.0066	0.0055	0.0053	0.1163	0.6595	0.0054	0.0060	0.0051
SdWgt	**0.0034**	0.0077	0.0047	0.0045	0.0154	0.5988	0.0037	0.0042	0.0035
Yield	0.0111	0.0147	0.0110	0.0116	0.0874	0.7241	0.0109	0.0115	**0.0107**

**Fig 4:**
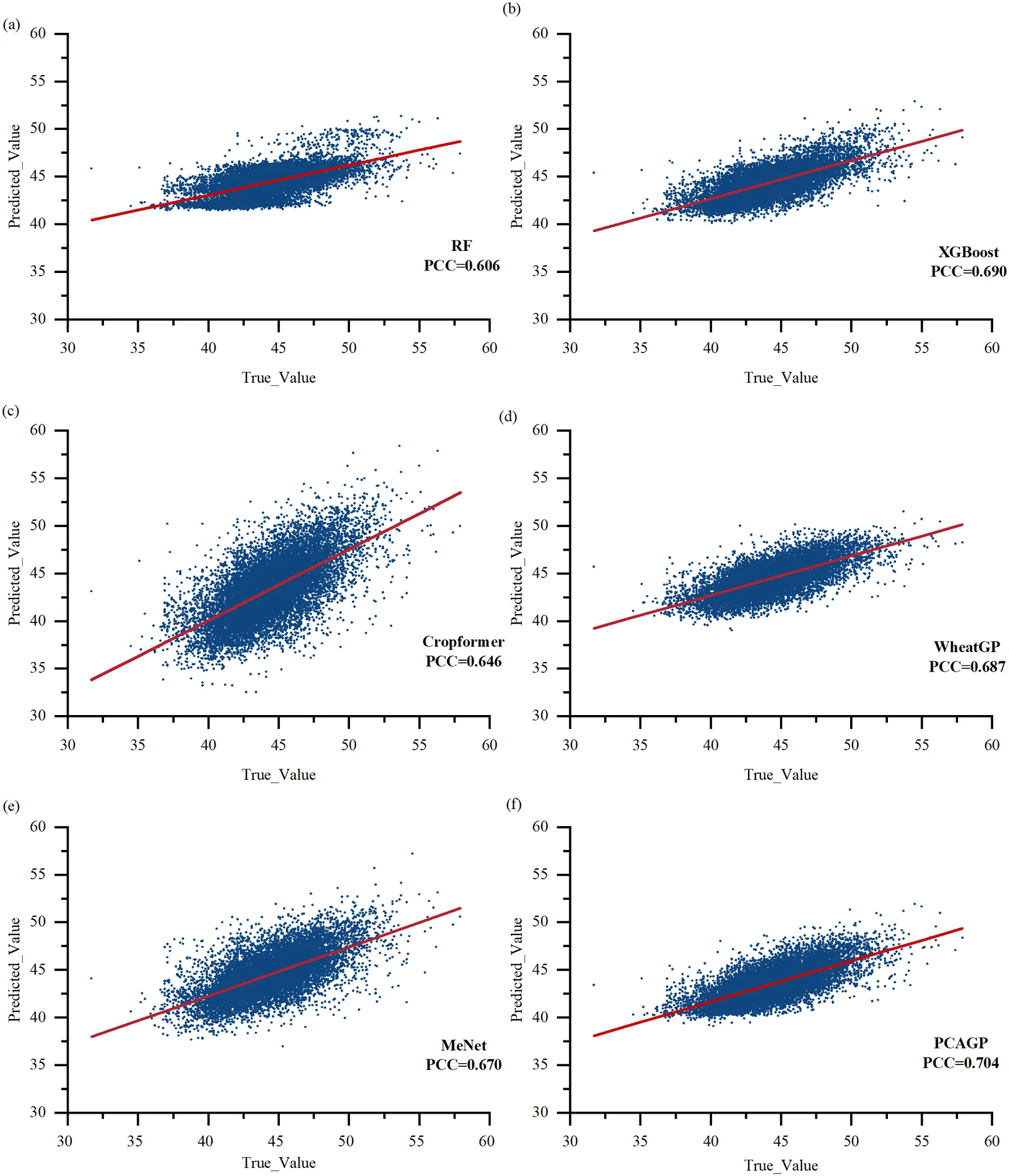
Scatter density plots of the prediction results of six methods for protein content in soybean13784. Trend lines describe linear regression. (a) RF; (b) XGBoost; (c) Cropformer; (d) WheatGP; (e) MeNet; (f) PCAGP.

To further evaluate the ability of our method to fit the distribution of actual observed data, we compared the prediction performance of six models for protein content in soybean13784 as shown in Fig 4. The scatter density plots depict the density distribution of predicted phenotypic values against their corresponding actual trait values. The trend lines, derived from linear regression fitting of the scattered points, indicate the correlation between model predictions and true phenotypic records. It can be observed that, compared with other prediction models, PCAGP exhibits a more clustered scatter distribution and lower dispersion, indicating that our method achieves higher prediction accuracy for most samples. Compared to MeNeT and Cropformer, PCAGP tends to narrow the prediction down to the average, with flatter trend lines and more conservative responses to extreme values. MeNeT and Cropformer, though yielding higher MSE, perform better at predicting extreme values such as high-yielding varieties. Thus, PCAGP sacrifices extreme‑value sensitivity for lower MSE and higher overall prediction accuracy while MeNeT and Cropformer achieve better extreme capture at the cost of higher MSE and lower overall prediction accuracy.

**Fig 5:**
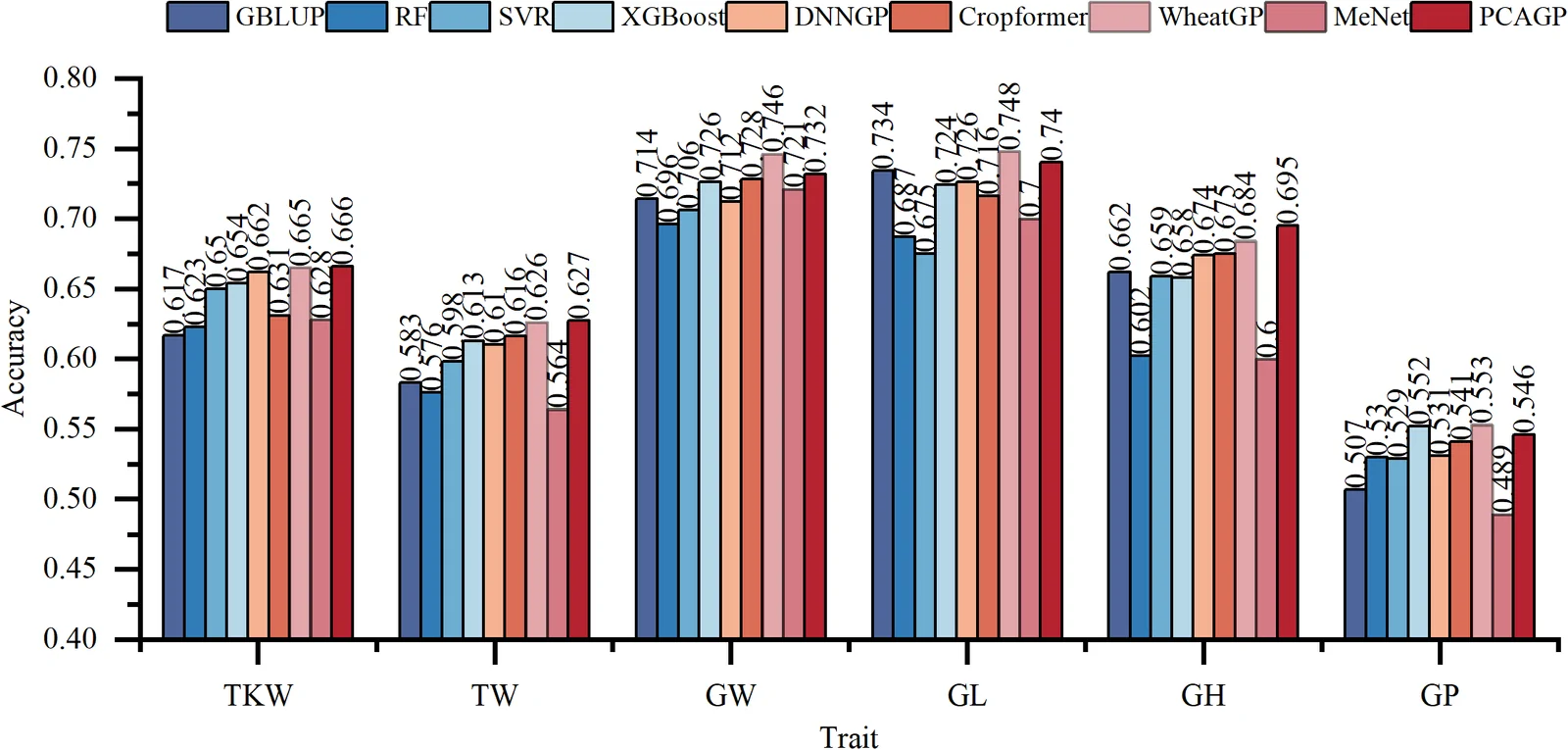
Prediction accuracy of nine methods on the wheat2000 dataset. TKW, TW, GW, GL, GH, and GP represent thousand kernel weight, test weight, grain width, grain length, grain hardness, and grain protein, respectively.

The prediction accuracy of different methods on the wheat2000 dataset are shown in Fig 5. It can be seen that WheatGP had the highest average prediction accuracy. WheatGP is a deep learning model specifically designed for Wheat, and it achieves the best prediction results on the wheat2000 dataset. PCAGP had a second-highest level of prediction accuracy, and outperformed GBLUP, RF, SVR, XGBoost, DNNGP, Cropformer, and MeNet by 5%, 7.9%, 5%, 2.1%, 2.4%, 2.5%, and 8.22%, respectively. The MSE values of different methods are shown in Table 3. PCAGP had the lowest average MSE value, 6.2%, 11.6%, 8.7%, 3.5%, 87.7%, 98.1%, 3.53%, and 15.51% lower than those of the comparison methods.

**Table 3:** MSE of Nine Methods on the Wheat2000 Dataset.

Trait	GBLUP	RF	SVR	XGBoost	DNNGP	Cropformer	WheatGP	MeNet	PCAGP
TKW	0.0260	0.0262	0.0248	0.0240	0.0958	0.9701	0.0244	0.0278	**0.0235**
TW	0.0134	0.0137	0.0138	0.0127	0.1727	1.4260	0.0129	0.0142	**0.0124**
GW	0.0099	0.0106	0.0106	0.0098	0.1339	0.7136	0.0104	0.0121	**0.0096**
GL	**0.0053**	0.0065	0.0071	0.0066	1.2235	0.0686	0.0057	**0.0053**	0.0055
GH	0.0206	0.0238	0.0216	0.0210	0.1022	1.0227	0.0198	0.0251	**0.0193**
GP	0.0125	0.0122	0.0123	**0.0116**	0.0927	0.8748	0.0118	0.0126	0.0117

**Fig 6:**
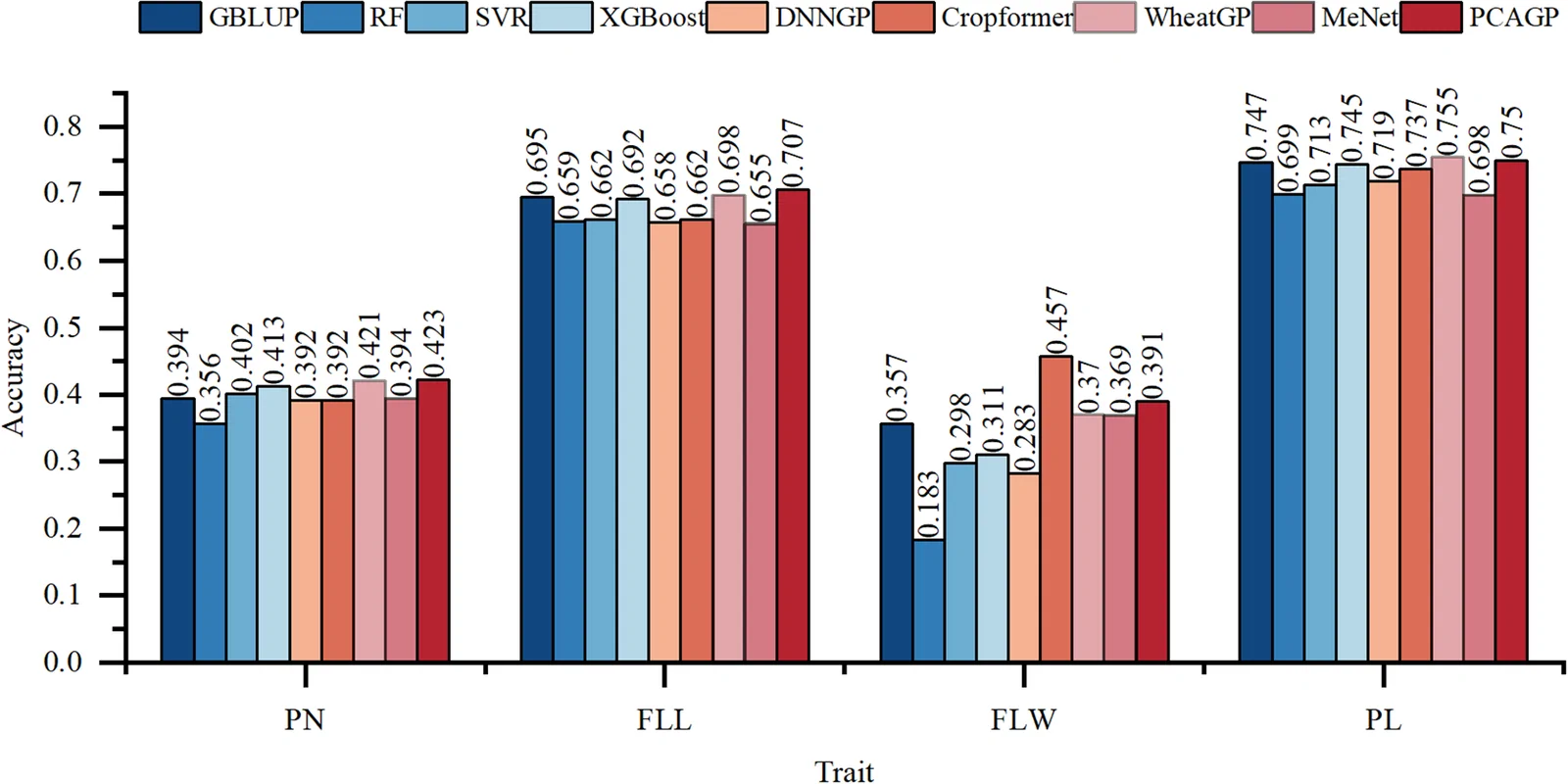
Prediction accuracy of nine methods on the rice1489 dataset. PN, FLL, FLW, and PL represent panicle number, flag leaf length, flag leaf width, and panicle length, respectively.

The prediction accuracy of different methods on the rice1489 dataset are shown in Fig 6 for PN, FLL, FLW, and PL. For all traits, PCAGP had the highest average prediction accuracy (0.568), and outperformed GBLUP, RF, SVR, XGBoost, DNNGP, Cropformer, WheatGP, and MeNet by 3.5%, 19.7%, 9.4%, 5.1%, 10.6%, 1%, 1.2%, and 7.33%, respectively. The MSE values of different methods are shown in Table 4. PCAGP had the lowest average MSE value, 1.1%, 11.9%, 24.6%, 3.3%, 41.4%, 99.2%, 4.32%, and 27.16% lower than hose of the comparison methods.

**Table 4:** MSE of Nine Methods on the Rice1489 Dataset.

Trait	GBLUP	RF	SVR	XGBoost	DNNGP	Cropformer	WheatGP	MeNet	PCAGP
PN	0.0118	0.0121	0.0120	0.0114	0.0162	1.4606	0.0115	0.0129	**0.0113**
FLL	0.0128	0.0144	0.0150	0.0131	0.0233	0.8347	0.0139	0.0196	**0.0127**
FLW	**0.0024**	0.0031	0.0090	0.0032	0.0030	1.1709	0.0025	0.0031	0.0025
PL	0.0090	0.0109	0.0110	0.0092	0.0183	0.9518	0.0091	0.0130	**0.0089**

The four datasets used in this study exhibit a significant variation in sample size, with the largest sample size consisting of 13784 individuals and the smallest having only 573 individuals. Although the sample size was relatively small, PCAGP still maintained high precision of genomic prediction, which demonstrated the advantages of PCAGP as a genomic prediction tool. In summary, compared to popular models such as GBLUP, RF, SVR, XGBoost, DNNGP, Cropformer, WheatGP, and MeNet, PCAGP demonstrates better prediction accuracy. In addition, PCAGP has good phenotype prediction accuracy on different crops.

### Effect of sample size on prediction methods

The selection of the size of the training population represents one of the most critical factors that affect the prediction accuracy. A reasonable size training population can capture the genetic variation of the target trait to the greatest extent, thus improving the reliability and stability of genomic prediction. To evaluate the impact of training sample size on prediction accuracy, we conducted comparisons on the wheat2000 [34] and rice1489 datasets [35]. For each dataset (six traits in wheat2000 and four traits in rice1489), we performed random sampling at five different proportions: 10%, 25%, 50%, and 75% of the full sample size, along with the complete dataset as reference. All experiments employed five-fold cross-validation, with final evaluation metrics representing averaged values across all folds.

**Fig 7:**
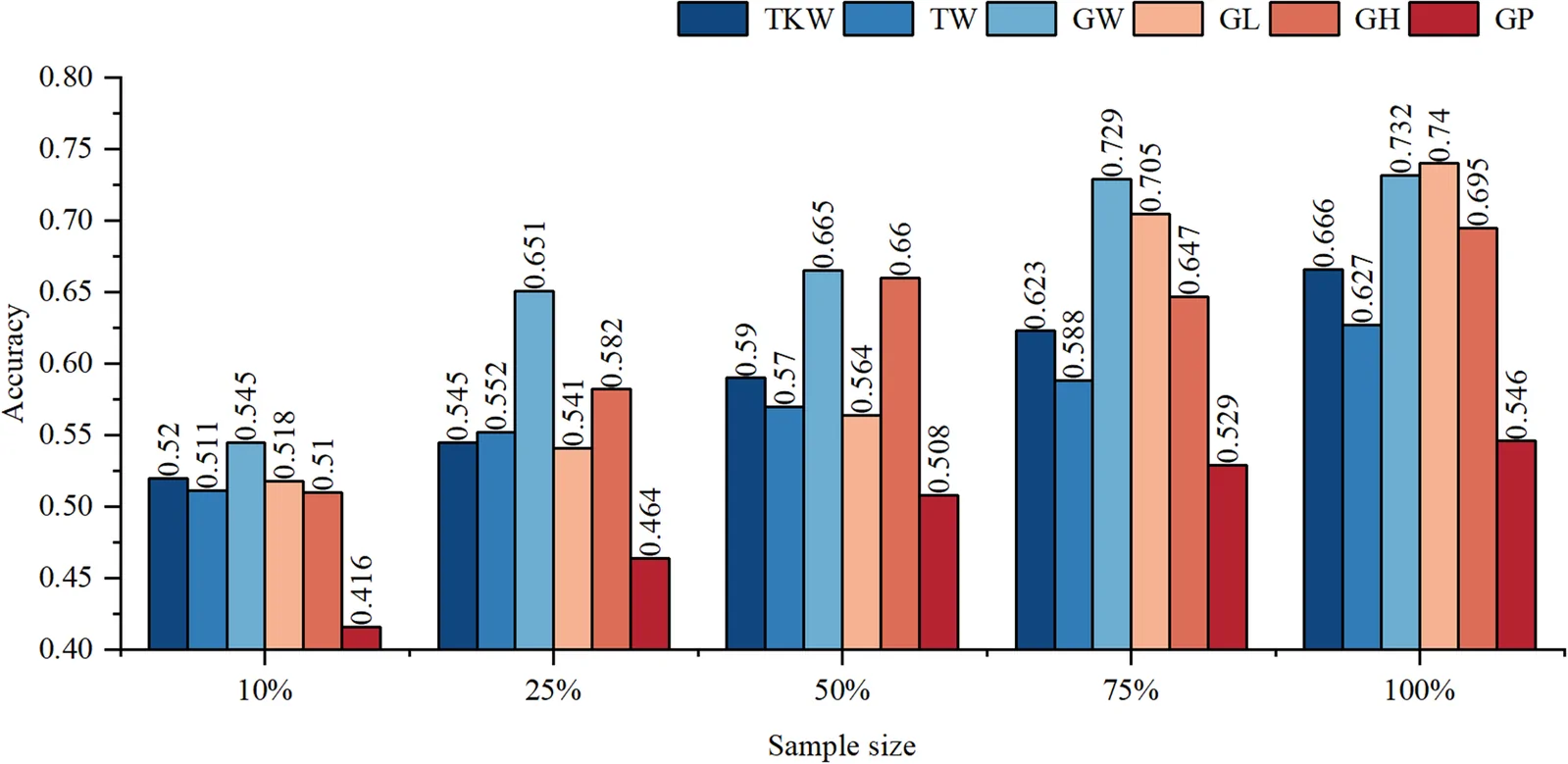
Prediction accuracy of PCAGP with different sample sizes on the wheat2000 dataset. TKW, TW, GW, GL, GH, and GP represent thousand kernel weight, test weight, grain width, grain length, grain hardness, and grain protein, respectively.

**Fig 8:**
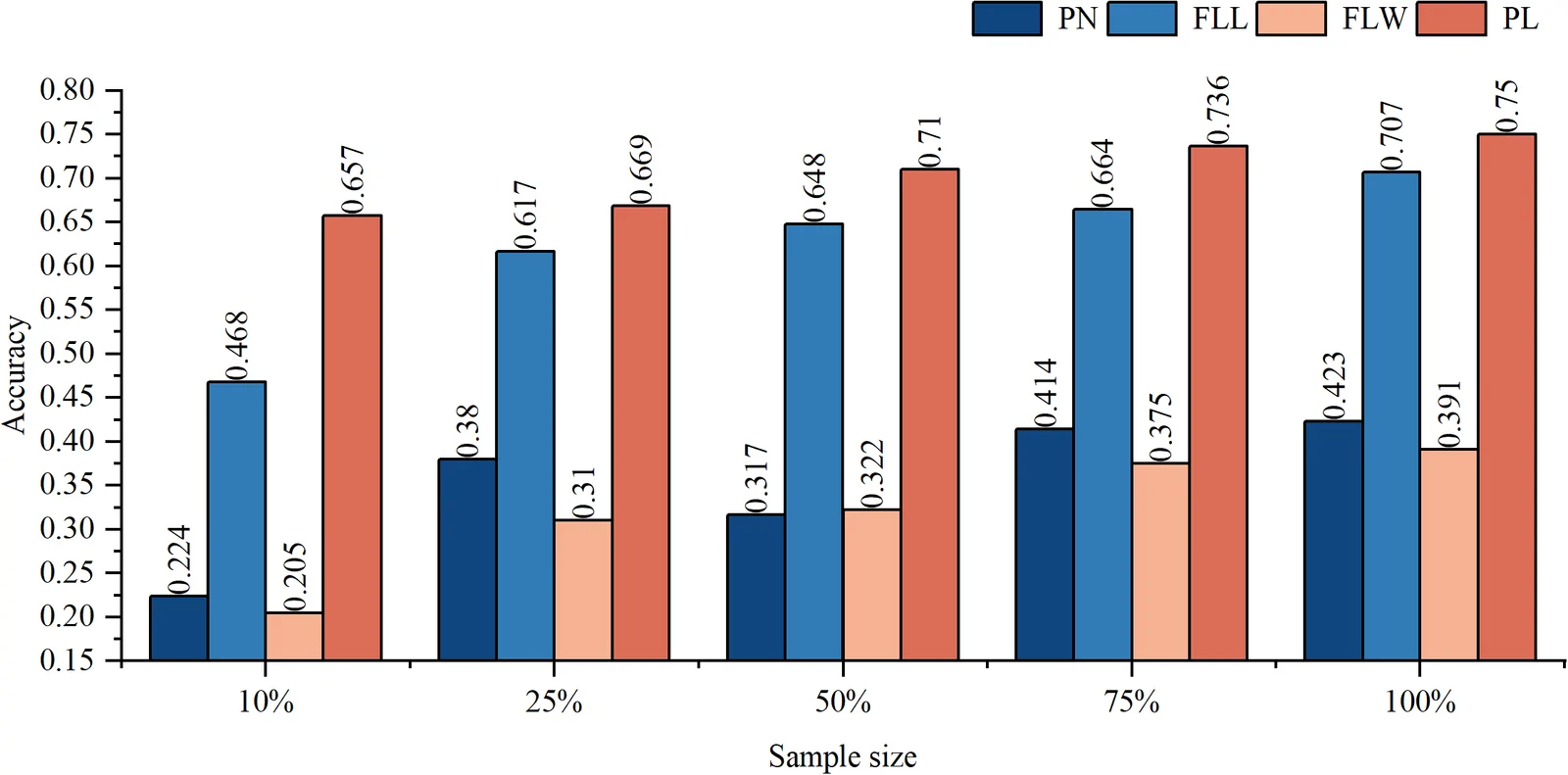
Prediction accuracy of PCAGP with different sample sizes on the rice1489 dataset. PN, FLL, FLW, and PL represent panicle number, flag leaf length, flag leaf width, and panicle length, respectively.

Fig 7 shows the prediction accuracy of PCAGP with different sample sizes on the wheat2000 dataset. When the sample size increased from 100 to 2000, the prediction accuracy of six traits TKW, TW, GW, GL, GH, and GP increased by 25.4%, 19.7%, 32.8%, 37.2%, 32.1%, and 31.4%, respectively. Fig 8 shows the prediction accuracy of PCAGP with different sample sizes on the rice1489 dataset. When the sample size increased from 148 to 1489, the prediction accuracy of six traits PN, FLL, FLW, and PL increased by 88.6%, 51.1%, 90.4%, and 14.2%, respectively. The MSE values of each trait corresponding to different sample sizes are shown in Table 5 and Table 6. With an increase in the sample size, the MSE values gradually decreased. Although the prediction accuracy of different traits decreased with the decrease in sample size, even with a small sample size of only 100, the average prediction accuracy of our model still exceeded 0.5. The results show that the PCAGP model can obtain good prediction performance even when the sample size is small.

**Table 5:** MSE of PCAGP with Different Sample Sizes on the Wheat2000 Dataset.

Trait	PCAGP(10%)	PCAGP(25%)	PCAGP(50%)	PCAGP(75%)	PCAGP(100%)
TKW	0.0411	0.0321	0.0286	0.0278	**0.0243**
TW	0.0174	0.0149	0.0148	**0.0126**	0.0130
GW	0.0167	0.0139	0.0137	0.0112	**0.0098**
GL	0.0097	0.0094	0.0081	**0.0063**	0.0069
GH	0.0311	0.0267	0.0235	0.0231	**0.0196**
GP	0.0177	0.0172	0.0134	0.0150	**0.0117**

**Table 6:** MSE of PCAGP with Different Sample Sizes on the Rice1489 Dataset.

Trait	PCAGP(10%)	PCAGP(25%)	PCAGP(50%)	PCAGP(75%)	PCAGP(100%)
PN	0.0126	0.0108	0.0126	0.0111	0.0129
FLL	0.0174	0.0138	0.0147	0.0129	0.0140
FLW	0.0007	0.0032	0.0021	0.0031	0.0025
PL	0.0110	0.0125	0.0103	0.0093	0.0102

### Effect of SNP number on prediction methods

A smaller number of SNPs that are significantly associated with target traits can achieve predictive performance similar to that of unfiltered genome-wide SNPs [19]. This is because the selected SNPs more accurately capture the genetic variation associated with the trait, improving the accuracy of the model’s prediction. To investigate the effect of SNP number on prediction accuracy, the wheat2000 dataset was used to evaluate the GBLUP, RF, DNNGP, and PCAGP models under varying P-value thresholds(P = 0.05, 0.01, 0.001, and 1e-04) for SNP selection. The number of SNPs retained under different P-value thresholds on the Wheat2000 dataset is shown in Table 7.

**Table 7:** The Number of SNPs Retained under Different P-Value Thresholds for Six Traits on the Wheat2000 Dataset.

Trait	*P* < 0.0001	*P* < 0.001	*P* < 0.01	*P* < 0.05	All SNPs
TKW	8424	10804	14304	17787	33709
TW	12173	14299	17215	20214	33709
GW	12992	15107	18021	21029	33709
GL	8319	10668	14051	17597	33709
GH	10695	12955	16105	19490	33709
GP	5041	7069	10383	14235	33709

**Fig 9:**
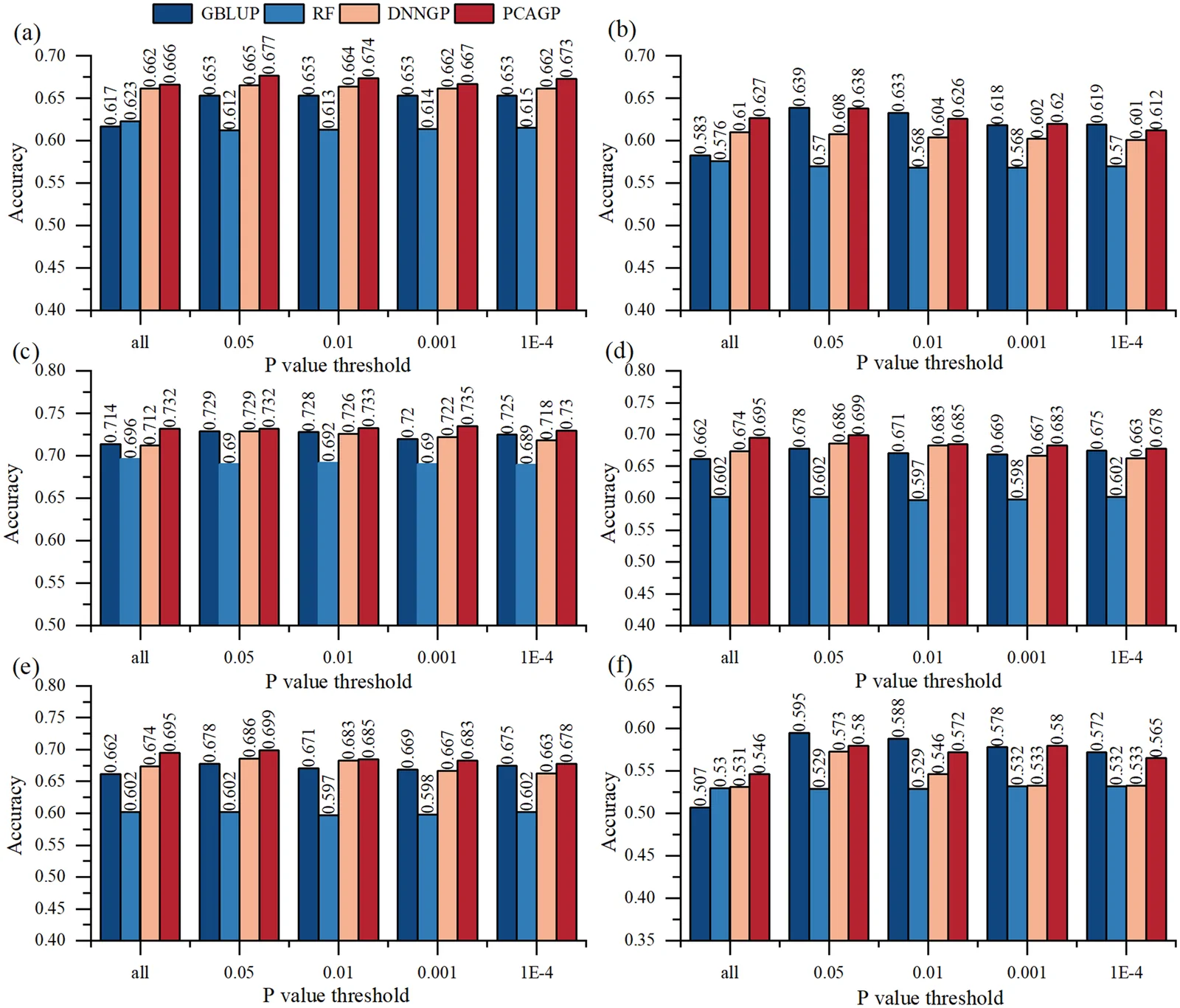
Prediction accuracy of four methods under various P-value thresholds on the wheat2000 dataset. (a) thousand kernel weight (TKW); (b) test weight (TW); (c) grain width (GW); (d) grain length (GL); (e) grain hardness (GH); (f) grain protein (GP).

The prediction accuracy and MSE of four methods under different P-value on the wheat2000 dataset are shown in Fig 9 and Table 8, respectively. We can see that the prediction accuracy and MSE of all methods do not change significantly as P-value screening became more rigorous. The results demonstrate that the use of trait-associated SNPs achieves predictive performance similar to that obtained by employing unfiltered genome-wide SNPs. Therefore, it is feasible to screen for SNPs using reasonable methods, so that the selected SNPs can reliably predict traits.

**Table 8:** MSE of Four Methods Under Various P-Value Thresholds on the Wheat2000 Dataset.

Trait	P-value threshold	GBLUP	RF	DNNGP	PCAGP
TKW	all	0.0261	0.0262	0.0958	**0.0235**
	0.05	0.0241	0.0265	0.0975	**0.0228**
	0.01	0.0241	0.0265	0.0881	**0.0222**
	0.001	0.0240	0.0264	0.0753	**0.0233**
	0.0001	0.0238	0.0263	0.0554	**0.0228**
TW	all	0.0134	0.0137	0.1727	**0.0124**
	0.05	0.0122	0.0138	0.1770	**0.0121**
	0.01	**0.0122**	0.0138	0.1685	0.0124
	0.001	**0.0123**	0.0138	0.1632	0.0129
	0.0001	**0.0126**	0.0138	0.1519	0.0127
GW	all	0.0106	0.0106	0.1339	**0.0096**
	0.05	**0.0095**	0.0107	0.1343	0.0096
	0.01	0.0096	0.0107	0.1261	**0.0095**
	0.001	**0.0092**	0.0107	0.1215	0.0094
	0.0001	**0.0090**	0.0113	0.1102	0.0091
GL	all	**0.0053**	0.0065	0.0686	0.0055
	0.05	**0.0050**	0.0065	0.0679	0.0053
	0.01	**0.0052**	0.0065	0.0605	**0.0052**
	0.001	**0.0052**	0.0065	0.0527	0.0055
	0.0001	**0.0050**	0.0063	0.0486	0.0053
GH	all	0.0206	0.0238	0.1022	**0.0193**
	0.05	0.0197	0.0238	0.0972	**0.0188**
	0.01	0.0201	0.0239	0.0922	**0.0194**
	0.001	0.0201	0.0239	0.0902	**0.0195**
	0.0001	0.0198	0.0238	0.0886	**0.0197**
GP	all	0.0125	0.0122	0.0927	**0.0117**
	0.05	**0.0107**	0.0120	0.0867	0.0114
	0.01	**0.0112**	0.0120	0.0701	0.0113
	0.001	**0.0111**	0.0121	0.0456	**0.0111**
	0.0001	**0.0112**	0.0121	0.0263	0.0114

In addition, reducing the number of input features also helps reduce computational complexity and mitigate the risk of overfitting. However, the effectiveness of this approach depends heavily on the accuracy of identifying SNPs that are truly associated with the target traits during the selection process. Improper selection may overlook important genetic signals, which adversely affects the predictive performance of the model. Thus, an appropriate screening method is essential to select a suitable number of SNPs that effectively represent the underlying genetic variation.

### Ablation experiments

To confirm the the effectiveness of integrating the parallel convolution with the coordinate attention mechanism, we performed ablation experiments using a parallel convolutional model without a coordinate attention. We also use different single convolutional layer with kernel sizes of 3 × 3, 5 × 5, and 7 × 7, respectively, to replace the parallel convolution layer.

The results of the PCAGP ablation experiments on the wheat2000 dataset are shown in Table 9. Compared with any single kernel convolution model or the model without attention mechanism, PCAGP shows better performance. From the perspective of the average prediction accuracy of each trait, PCAGP outperformed the models using a single convolution layer with kernel sizes of 3 × 3, 5 × 5, and 7 × 7, as well as the model using only the parallel convolution structure, by 5.6%, 4.6%, 4.3%, and 2.9% respectively. The model can capture local details and global context features at the same time, and enhance spatial perception and location sensitivity through the attention mechanism, thus significantly improving the feature expression ability and prediction accuracy. Therefore, the combination of the parallel convolution structure and the coordinate attention mechanism not only has structural rationality, but also shows significant advantages in performance, which verifies its effectiveness and necessity in this task.

**Table 9:** Prediction Accuracy Comparison of Different Kernel Sizes and CA Module Configurations.

Trait	3 × 3	5 × 5	7 × 7	Parallel convolution	PCAGP
TKW	0.6400	0.6343	0.6399	0.6533	**0.6664**
TW	0.5853	0.5973	0.5962	0.6008	**0.6271**
GW	0.7018	0.7028	0.7067	0.7182	**0.7318**
GL	0.6873	0.7016	0.7064	0.7086	**0.7397**
GH	0.6520	0.6588	0.6599	0.6720	**0.6954**
GP	0.5271	0.5357	0.5311	0.5401	**0.5461**

The number of parameters of five deep learning methods are compared, as shown in Table 10. It can be seen that significant differences exist in the model complexity among different prediction methods. MeNet has the largest number of parameters, which is 71.6 million. Its network structure is relatively complex and the computational overhead is relatively high. In contrast, PCAGP has only 1.38M of parameters, which is the smallest number among the five methods. Parallel computing techniques can also potentially accelerate the computation of parallel convolutions. Therefore, PCAGP can achieve trait prediction with low complexity, thereby reducing computational cost and achieving high prediction performance.

**Table 10:** The Number of Parameters of Five Deep Learning Methods.

Method	Parameter quantity
DNNGP	3.07M
Cropformer	3.87M
WheatGP	25.1M
MeNet	71.6M
PCAGP	**1.38M**

## Discussion

The innovation of theoretical methods has laid the foundation for the development of GS technology. However, to truly apply it to breeding, efficient prediction of crop genotypes and phenotypes is essential. There is an urgent need to develop GS methods with greater prediction accuracy. Although models such as GBLUP [9], RF [36], SVR [37], XGBoost [15], DNNGP [19], Cropformer [21], WheatGP [24], and MeNet [22] have made significant contributions to this field, they also exhibit certain limitations. In particular, the classical deep learning models all use a serial structured convolution kernel to extract features. Although it is possible to explore the complex relationships in SNPs to some extent, determining the size of the convolution kernels is also a very time-consuming task. Moreover, the serial structure may also have the risk of information loss. This loss of information can impair the model’s ability to capture intricate genetic interactions and their influence on phenotypic traits, particularly in highly nonlinear genomic data.

In this paper, a method based on a parallel convolutional attention network for crop genomic prediction (PCAGP) was constructed, through which the relationships among genotype data can be effectively learned via parallel convolutions to predict phenotypes. Benchmarking on multiple crop datasets shows that PCAGP outperforms existing GS models, with higher overall accuracy, lower MSE, and lower computational cost. The main advantages of PCAGP are summarized as follows.

Firstly, the PCAGP model adopts parallel convolutional layer simultaneously to extract features, which offers significant computational efficiency advantages. Compared with serial convolutional layers, parallel processing can be accelerated by using parallel computing technology, improving computational efficiency, and thus enabling faster analysis of large-scale data.

Secondly, the PCAGP model introduces a coordinate attention module, which ensures that the genotype data features extracted through parallel convolution are assigned different weights based on the correlation of channel and spatial positional information. This enables the model to exhibit higher prediction accuracy.

Finally, the overall design of the PCAGP model does not rely on deep network layers. In phenotypic prediction, the number of SNPs is much greater than the number of samples, and the use of deep network layer may lead to overfitting. A wider network layer is more beneficial for feature representation, while minimizing information loss and preserving more data details.

## Conclusion

This paper introduces a novel genome-wide prediction method called PCAGP. The proposed model transforms the SNP data of each sample into a two-dimensional matrix representation. By employing a parallel convolutional architecture with kernels of varying sizes, PCAGP enables more comprehensive and efficient genotype feature extraction. Furthermore, the integration of a coordinate attention mechanism allows the model to accurately capture positional information while effectively modeling long-range dependencies among SNPs. Experimental results demonstrate that our model exhibits strong generalization ability across diverse crop species for agronomic trait prediction tasks, consistently achieving high prediction accuracy. These findings validate the robustness and effectiveness of the PCAGP model, suggesting its broad potential for application in GS research and modern breeding programs.
